# New Horizons in *KRAS*-Mutant Lung Cancer: Dawn After Darkness

**DOI:** 10.3389/fonc.2019.00953

**Published:** 2019-09-25

**Authors:** Haitang Yang, Shun-Qing Liang, Ralph A. Schmid, Ren-Wang Peng

**Affiliations:** ^1^Department of General Thoracic Surgery, Department of BioMedical Research, Inselspital, Bern University Hospital, University of Bern, Bern, Switzerland; ^2^University of Massachusetts Medical School, Worcester, MA, United States

**Keywords:** lung cancer, *KRAS*, mitogen-activated protein kinases, heterogeneity, targeted therapy, immunotherapy

## Abstract

In non-small cell lung cancer (NSCLC), the most frequent oncogenic mutation in western countries is *KRAS*, for which, however, there remains no clinically approved targeted therapies. Recent progress on high biological heterogeneity including diverse *KRAS* point mutations, varying dependence on mutant *KRAS*, wide spectrum of other co-occurring genetic alterations, as well as distinct cellular status across the epithelial-to-mesenchymal transition (EMT), has not only deepened our understanding about the pathobiology of *KRAS*-mutant NSCLC but also brought about unprecedented new hopes for precision treatment of patients. In this review, we provide an update on the most recent advances in *KRAS*-mutant lung cancer, with a focus on mechanistic insights into tumor heterogeneity, the potential clinic implications and new therapies on horizons tailored for *KRAS*-mutant lung cancer.

## Introduction

Lung cancer is the most common cancer with high lethality (1). Carcinogenic Kirsten rat sarcoma viral oncogene homolog (*KRAS*) mutation is the most common gain-of-function alteration, accounting for ~30% of lung adenocarcinomas in western countries and about 10% of Asian lung adenocarcinomas (2).

As a membrane-bound small GTPase, KRAS switches between the active GTP-bound and inactive GDP-bound status, which is regulated by guanine nucleotide exchange factors (GEFs) and GTPase-activating proteins (GAPs), respectively (3). The intrinsic GTPase activity of RAS is rather low, but in the presence of GAPs, such as neurofibromin 1 (NF1), its hydrolytic activity can be increased by several orders of magnitude. Reactivation of GDP-bound RAS is mediated by GEFs, such as son of sevenless homolog 1 (SOS1), which promotes the release of bound GDP, and then cellular GTP will replace GDP to bind to RAS. Carcinogenic mutations impair the ability of KRAS to hydrolyze GTP and are thought to lock the oncoprotein in a constitutively active state by activating KRAS downstream signaling cascades, leading to uncontrolled cell proliferation and survival. In patients with lung cancer harboring *KRAS* mutations, the most mutations occur in codon 12, whereas mutations in codons 13 and 61 are less frequent (4).

In lung cancer, considerable progress in developing molecularly-driven therapeutics has been made in the past decades, mainly including targeted therapies against oncogenic drivers, such as *EGFR, HER2, EML4-ALK, MET, ROS1*, and *BRAF* mutations, and immunotherapies in non-oncogene-driven lung cancer, such as PD1 and PDL1 alterations (5, 6). However, for *KRAS*-mutant lung cancer, the treatment options are still limited, and chemotherapies remain the first-line recommendation.

In this review, we update the recent clinically relevant aspects of the pathobiology of *KRAS*-mutant non-small cell lung cancer (NSCLC), mainly focusing on tumor heterogeneity, therapeutic implications, and new treatment opportunities.

## Heterogeneity in *KRAS*-Mutant Lung Cancer

### Diverse Point Mutations in *KRAS*

**Figure 1:**
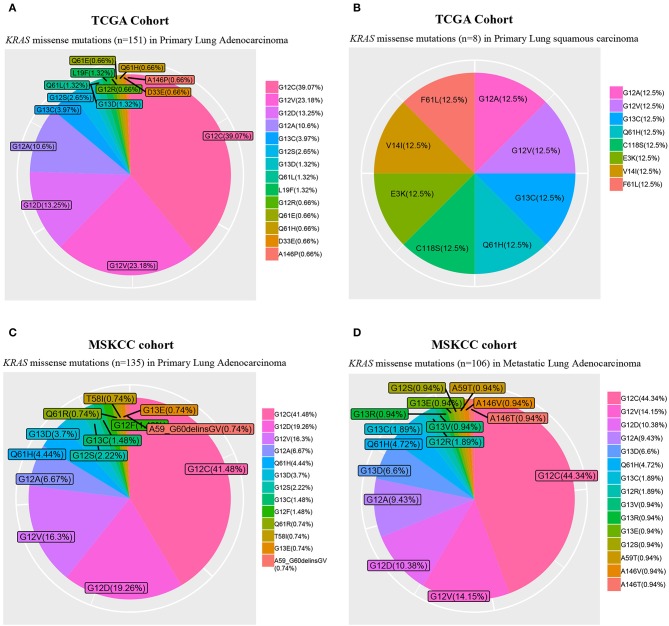
Frequency of *KRAS* missense mutations. The Cancer Genome Atlas (TCGA) primary lung adenocarcinoma (*n* = 489; **A**) and squamous cell carcinoma (*n* = 492; **B**) cohorts, and Memorial Sloan Kettering Cancer Center (MSKCC) primary (*n* = 471; **C**) and metastatic lung adenocarcinoma (*n* = 444; **D**) cohorts.

In lung cancer, *KRAS* mutations occur primarily in adenocarcinoma, whereas they are only rarely seen in squamous cell carcinoma (Figure 1). Diverse point mutations exist within *KRAS*, the majority of which affect codon 12 of the protein in NSCLC (Figure 1), leading to amino acid substitutions that impair the intrinsic hydrolytic activity and render the KRAS oncoprotein constitutively active.

In lung cancer, the presence of KRAS amino acid substitution influences patients' prognosis and is negatively associated with patient response to targeted therapy (7, 8) and chemotherapy (9–11). Molecular modeling studies showed that different conformations imposed by distinct *KRAS* oncogene substitutions could lead to altered association with downstream signaling transducers (12). Specifically, compared to wild-type *KRAS*, the mutant *KRAS*^G12C^ or *KRAS*^G12V^ is less dependent on AKT, which, however, is more intimately engaged by other mutant KRAS proteins.

Mutant KRAS with different amino acid substitutions may also associate with distinct biological behavior (13) and can lead to different clinical outcomes (14–16). In *KRAS*-mutant lung cancer, tumors carrying *KRAS*^G12C^ exhibited higher ERK1/2 phosphorylation than those with *KRAS*^G12D^ (17). In supporting this observation, studies with genetically engineered mouse model showed that *Kras*^G12C^ tumors were significantly more sensitive to MEK inhibitor than the *Kras*^G12D^ ones and that MEK inhibition significantly increased chemotherapeutic efficacy and progression-free survival (PFS) of *KRAS*^G12C^ mice.

Taken together, different amino acid substitutions in oncogenic KRAS lead to heterogeneity in biological behaviors of the mutant protein, implying the need of genotype-specific analysis to identify clinically relevant subgroups of patients that may ultimately influence treatment decisions. It also should be taken into account for different downstream signaling pathways to be inhibited for patients with tumors carrying different KRAS amino acid substitutions.

### KRAS Dependence Score and EMT

The concept of *KRAS* dependence or independence was proposed based on the observations that in both patients and cell lines, tumors frequently exhibit unexplained intrinsic resistance to *KRAS*-targeted therapy, by either inhibitors or genetic ablations. Mutant *KRAS* has been considered as an oncogenic driver. However, whether it is indispensable in each tumor carrying this oncogene is not clear. Early evidence suggested that not all *KRAS*-mutant tumor cells are dependent on KRAS (18, 19), and that some *KRAS*-mutant cancer cells, including lung (20) and pancreatic cancer cells (21, 22), can survive in the absence of the *KRAS* oncogene. These observations provide additional layers of evidence that make targeting *KRAS*-mutant tumors more complex.

Oncogenic KRAS can activate various downstream effector pathways, and the best characterized are phosphatidylinositol 3-kinase (PI3K) and mitogen-activated protein kinases (MAPK) (23–25). Oncogenic *KRAS* signaling proceeded by different downstream effectors may lead to phenotypic variance in cancer, but to what extent the downstream effectors contribute to the oncogenic phenotype is not fully understood. Recently, Yuan et al. designed a combinatorial siRNA-based approach to functionally discern the link between KRAS downstream effectors and phenotypic variation in a large panel of cancer cell lines, and identified two major subtypes within *KRAS*-mutant cancers based on the dependence on KRAS or RSK (Ribosomal Protein S6 Kinase A1) (25). Interestingly, besides the distinct morphologies and effector landscapes, the two subtypes also differ in metabolic status with therapeutically tractable vulnerabilities. The heterogeneity in effector signaling pathways across *KRAS*-mutant cells presents a significant challenge to identify universal synthetic partners lethal to mutant *KRAS*.

It is well-documented that the epithelial-to-mesenchymal transition (EMT) process is closely related to therapy resistance. Interestingly, *KRAS*-mutant cancer cells dependent on or addicted to *KRAS* oncogene are more associated with an epithelial phenotype, whereas those independent of *KRAS* adopt a mesenchymal phenotype (18). Importantly, *KRAS*-mutant cancer cells differing in EMT status vary in their responses to MEK inhibitors (26), as EMT rewires the expression of receptor tyrosine kinases (RTKs), a consequence of differential feedback activation of the MAPK pathway following MEK inhibition. In epithelial-like cancer cells, ERBB3 is preferentially activated by feedback signaling, which reactivates MEK and AKT signaling. In mesenchymal-like *KRAS*-mutant cancer cells, reactivation of MEK and AKT was dominantly driven by FGFR1. Signaling transduced by FGFR is normally suppressed by the sprouty proteins (SPRY4), but MEK inhibition represses the negative regulation of SPRY4. In line with this, another independent study using short hairpin (sh) RNA screen had similar findings in *KRAS*-mutant lung and pancreatic cancer cells (27). These findings provide a strong therapeutic rationale to treat epithelial *KRAS*-mutant lung cancer (high epithelial markers) with clinically available ERBB and MEK inhibitors, and mesenchymal-like *KRAS*-mutant lung cancers (high FGFR1) by combined therapy with FGFR and MEK inhibitors.

The association of tumor response to MEK inhibitor therapy to EMT status of cancer cells was further investigated by a more recent study (28). Peng et al. identified an inverse correlation between MAPK signaling dependency and a zinc finger E-box binding homeobox 1 (ZEB1)–mediated EMT in patient samples harboring *KRAS, BRAF*, or *NRAS* mutations. Mechanistic results indicated that MAPK dependency is dictated by the functional interplay between scaffold protein interleukin-17 receptor D (IL17RD) and ZEB1. Mechanistically, in mesenchymal-like *KRAS*-mutant lung cancer cells, ZEB1 directly represses IL17RD to mediate the resistance to MEK inhibitors. Based on this, ZEB1 suppression by miR-200 expression or histone deacetylase inhibitor (mocetinostat) re-sensitized mesenchymal cells to MEK inhibition and markedly reduced *in vivo* tumor growth. This study provided the mechanistic support for combinatorial treatment (MEK plus histone deacetylase inhibitors) for *KRAS*-mutant lung cancer, and, again, highlighted the importance of stratification of epithelial and mesenchymal subsets in decision-making for treating *KRAS*-mutant lung cancer.

### Genetic Alterations Co-occurring With KRAS Mutations

**Figure 2:**
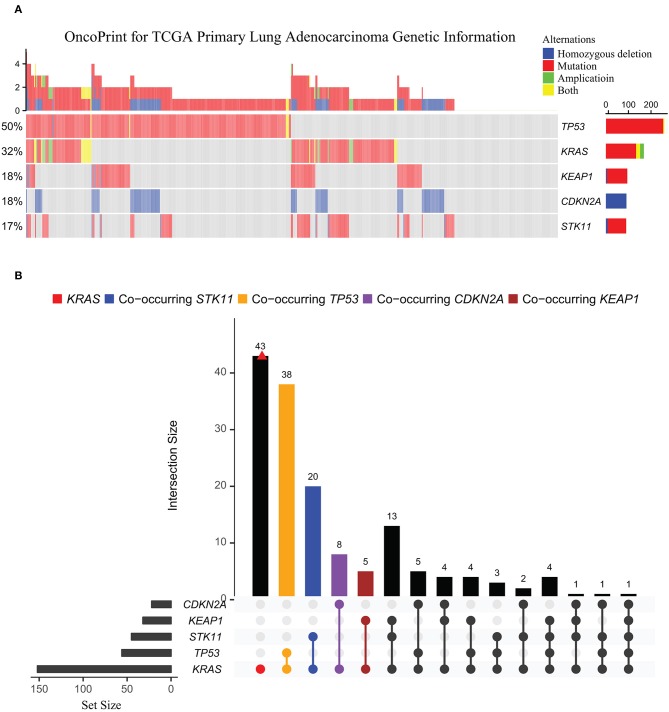
Oncoprint for common genetic alterations co-occurring with *KRAS* in TCGA lung adenocarcinoma cohort. **(A)** The genetic landscape of *KRAS, TP53, KEAP1, CDKN2A*, and *STK11* in TCGA primary lung adenocarcinoma cohort (*n* = 489). **(B)** UpSetR plot visualizing the intersections of other mutations co-occurring with *KRAS*-mutant across TCGA lung adenocarcinomas. The intersection points are indicated by different colors: *CDKN2A* (purple), *KEAP1* (brown), *STK11* (blue), and *TP53* (orange). Intersections among the co-occurring genes were connected with a line, with frequency of each co-occurring mutation type shown on the bar plot. Set metadata is shown to the left (charts).

Heterogeneity in *KRAS*-mutant tumors also arises from co-occurring alterations of other genes, e.g., *TP53, CDKN2A/2B, STK11*, and *KEAP1* (Figure 2). Compelling evidence showed that co-occurring genomic changes could profoundly affect biological behaviors (29–31), clinical outcomes (32), and therapeutic vulnerabilities of *KRAS*-mutant cancers (33, 34).

An integrative study of genomics, transcriptomics, and proteomics in early-stage and chemo-refractory *KRAS*-mutant lung adenocarcinomas identified three major subsets defined by co-occurring genetic alterations in *STK11/LKB1* (KL subgroup), *TP53* (KP subgroup), and *CDKN2A/B* (KC subgroup) (29). The three subgroups differ in biological properties and therapeutic vulnerabilities, with KC tumors associated with suppressed mTORC1 signaling and KL tumors with lower expression of immune markers (e.g., PD-L1) if *KEAP1* co-mutated, while higher levels of somatic mutations, inflammatory and immune checkpoint markers, and prolonged relapse-free survival were observed in KP tumors. Further, KL cells exhibited heightened vulnerability to HSP90 inhibition. This work argued that genomic alterations co-occurring with mutant KRAS stratify lung adenocarcinomas and define pathobiological properties and therapeutic vulnerabilities.

A more recent study of a large patient cohort (*n* = 330) with advanced *KRAS*-mutant lung cancer identified co-mutated *KEAP1* as an independent prognostic marker for poorer survival [HR = 1.96; *P* < 0.001] and as being associated with less response to chemotherapy [HR = 1.64; *P* = 0.03] and immune therapy [HR = 3.54; *P* = 0.003] (30). Another study showed that presence of co-mutated *Trp53* reduces sensitivity to combined treatment with MEK inhibitor and chemotherapy in *Kras*^G12C^-driven murine lung cancer, which supports further clinical investigations of the combination therapy for patients with lung cancer harboring *KRAS*^G12C^ and wild-type p53 (17). Finally, yet importantly, *STK11/LKB1* alterations have been described as a major driver of primary resistance to PD-1 blockade in *KRAS*-mutant lung adenocarcinoma (31, 35).

Supporting this, a recent study (36) showed that among 377 non-squamous NSCLC patients treated with platinum-doublet chemotherapy (carboplatin or cisplatin and pemetrexed) plus pembrolizumab (anti-PD1), the therapy response was significantly associated with the genetic status of *STK11*. Specifically, patients with genomic alterations of *STK11* (*N* = 102) were associated with significantly shorter PFS (4.8 vs. 7.2 months, HR 1.5, 95% CI 1.1–2.0; *P* = 0.0063) and shorter overall survival (10.6 vs. 16.7 months, HR 1.58, 95% CI 1.09–2.27; *P* = 0.0083) compared with patients without *STK11* alteration (*N* = 275). Also, the objective response rate (RR) was significantly different between the two groups (32.6 vs. 44.7%, *P* = 0.049). More importantly, the addition of pembrolizumab to platinum-doublet chemotherapy did not significantly improve PFS (4.8 vs. 4.3 months, HR 1.13, 95% CI 0.83–1.54, *P* = 0.75) or overall survival (10.6 vs. 10.3 months, HR 1.03, 95% CI 0.71–1.49, *P* = 0.79) compared to the chemotherapy alone (36). This study defines a subgroup of patients with *STK11* alterations who do not benefit from immunotherapy, indicating the importance of cancer genetic information for stratification of patients who would benefit from immune checkpoint blockade. Apparently, co-occurring alterations further increase the heterogeneous complexity, which may explain inconsistent outcomes of clinical trials with *KRAS*-mutant lung cancers.

## New Horizons for Treating *KRAS*-Mutant Lung Cancer

### Refocusing on Direct Targeting of KRAS

For decades, KRAS was considered undruggable due to its high affinity for GTP and the lack of a clear binding pocket. Enormous attempts and efforts had been made, but all failed to identify compounds that could effectively and directly target mutant RAS. Since then, there has been little advance. However, with new technologies in drug development and novel mechanistic insights into RAS biology, attention has been refocused on the approach that directly interferes with the function of RAS oncoproteins, with more effort given to find the way to target mutant alleles specifically.

Earlier studies have identified small molecules selectively recognizing and irreversibly inactivating one specific *KRAS*-mutant allele harboring a G12C amino acid substitution (37, 38). A breakthrough of direct RAS targeting was finally made by Ostrem et al., who, by using a novel screening technology called tethering, developed a new strategy to target mutant *KRAS*^G12C^ specifically without affecting the wild-type protein (37). This work also suggested that the previous perception of mutant KRAS was persistently locked in its active GTP-bound state might not be true.

Later on, Lim et al. reported the synthesis of a GDP analog, SML-8-73-1, which contains an electrophilic chloroacetamide attached to the β-phosphate. This analog can covalently modify cysteine 12 of KRas^G12C^ and, as a result, it competes with GTP and GDP for active site binding in a cellular context (38). Despite the pioneering development of the KRAS^G12C^-specific inhibitors, follow-up studies indicated that these initial compounds showed only limited potency (39, 40). In a search for more effective compounds or analogs, ARS853 was developed (40), which selectively reduced KRAS-GTP levels by more than 90% and increased the *in vitr*o hydrolytic reaction rate by 600-fold compared to the initial compound used in Ostrem et al. (37). At the micromolar range, ARS853 potently suppressed MAPK and PI3K-AKT signaling. Thus, KRAS^G12C^ mutant protein is in a dynamically rather than a statically active state and targeting the inactive, GDP-bound form of KRAS is a realistic and promising anti-RAS therapeutic. These striking findings were recently translated into *in vivo* studies, in which a new covalent KRAS^G12C^-specific inhibitor, ARS-1620, showed rapid and durable tumor regression in mice (41).

These studies prompt a revisit to target KRAS oncoproteins directly. Recent discoveries have enabled further development and investigation of more compounds of this family in clinical trials (Table 1). Encouraging phase I clinical trial data of AMG510 (Amgen, clinical trial information: NCT03600883) in 32 patients with *KRAS*^G12C^ mutation (14 with NSCLC, 19 with colorectal cancer, and 2 with appendix cancer) were just released in ASCO 2019. Five of 10 evaluable patients with NSCLC had a partial response, and four had stable disease, in total achieving a disease control rate of 90% (9/10). Additionally, 13 of 18 evaluable patients with colorectal cancer experienced stable disease. Twenty-six patients were still under study, and nine discontinued. Importantly, the treatment was well-tolerated, with primarily grade 1 events (68%). Two grade 3 treatment-related AEs were reported (anemia and diarrhea). No grade 4 or more severe treatment-related adverse effects were reported. MRTX849 is another potent, highly selective, and orally available small-molecule inhibitor of KRAS^G12C^ (42). MRTX849 shows broad-spectrum anti-tumor activity in a panel of patient- and cell-derived *in vivo* tumor models with KRAS G12C-substitution, with complete tumor regression observed in a subset of these models.

**Table 1:** Ongoing clinical trials involving direct targeting of KRAS.

**Compounds**	**Company**	**Mechanism**	**Clinical trial**
AMG 510	Amgen/Carmot Therapeutics	KRAS^G12C^ inhibitor	NCT03600883
MRTX849	Mirati (ex Array)	KRAS^G12C^ inhibitor	NCT03785249
KRAS TCR	Gilead (ex Kite/NCI)	Anti-KRAS^G12D^ engineered T-cell receptor	NCT03745326
AZD4785	AstraZeneca/Ionis	KRAS antisense oligonucleotide	NCT03101839

Different from the inhibitors that directly target mutant KRAS, AZD4785 is a *KRAS* antisense oligonucleotide that targets the *KRAS* gene irrespective of its mutation status (43). Despite AZD4785 being safe and well-tolerated, the first phase I trial failed, which might be due to the fact that AZD4785 targets both mutant and wild-type *KRAS* mRNA for degradation.

Tran and colleagues described a case of a patient with metastatic colorectal cancer treated with autologous T cells specific for mutant KRAS^G12D^, which was restricted to the major histocompatibility complex class I allele HLA-C^*^08:02 (44). Despite the rarity of HLA-C^*^08:02, this study demonstrated the promise of T-cell–based immunotherapy for targeting KRAS^G12D^ and HLA-C^*^08:02. Further evaluation in more patients is warranted.

Whether direct inhibition of KRAS with these new compounds is sufficient remains a question, given the presence of KRAS independence in tumor cells harboring *KRAS* mutations. Concurrent inhibition of collateral dependencies may be required to potentiate the effectiveness of those compounds.

### Reinforcing MEK Inhibitors

**Figure 3:**
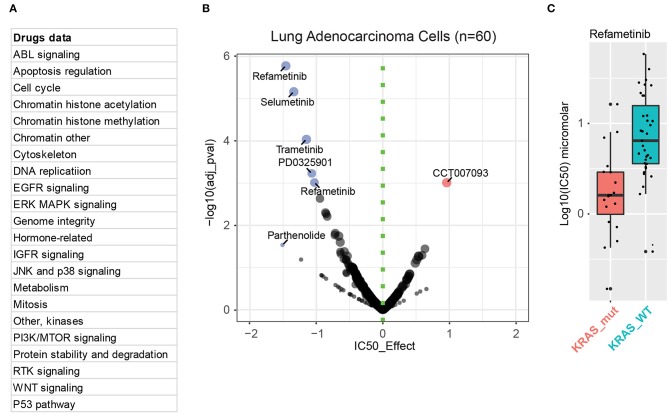
MEK mediates the key downstream effect in *KRAS*-mutant lung cancer cells. **(A)** Drug information incorporated in the Genomics of Drug Sensitivity in Cancer database. **(B)** Integrative analysis of drug sensitivity data of *KRAS*-mutant (*n* = 20) lung adenocarcinoma cells compared to *KRAS*-wild-type (WT; *n* = 40) ones. In the volcano plot, the x-axis indicates the IC50 effect, with effect < 0 representing *KRAS*-mutation sensitive inhibitors (in blue) compared with *KRAS*-WT ones. The color intensity and the circle size are proportional to significance value (the y-axis). **(C)** Sensitivity analysis of *KRAS*-mutant (in red) and WT (in blue) cells to one of the MEK inhibitors, refametinib.

To date, most efforts to treat cancers with *RAS* mutations have focused on targeting downstream effectors of mutant RAS, such as RAF, MEK, or PI3K, each of which is druggable. Although, as described above, different KRAS mutations show a preference for activating different downstream signaling, hyperactivation of the mitogen-activated protein kinase (MAPK) pathway is generally recognized as a key feature in *KRAS*-driven lung cancer cells. One reason is that the G12C substitution (44%), the most common subtype in *KRAS*-mutant lung cancer, shows more prominent engagement with MAPK signaling. Supporting these findings, we performed pooled drug sensitivity analysis based on publicly available dataset in Genomics of Drug Sensitivity in Cancer, which revealed that, compared with *KRAS*-wild-type lung cancer cells, *KRAS*-mutant lung cancer cells are exclusively more sensitive to various MEK inhibitors rather than those targeting other oncogenic pathways (Figure 3). This explains why MEK inhibitors have been the most widely investigated, typically as a combination therapy, despite the presence of multiple inhibitors that are being explored to target different KRAS-activated pathways.

#### Rethinking Combination Treatment With Chemotherapy

In the clinic, conventional chemotherapy is widely used to treat patients with *KRAS*-mutant NSCLC, although chemotherapy plus immune checkpoint blockade has been recently approved as the first-line regimen for NSCLC, including patients with *KRAS* mutations (45, 46). Clinical trials evaluating the efficacy of selumetinib, a potent MEK inhibitor, to potentiate chemotherapy, have recently been conducted.

A survival benefit of selumetinib plus docetaxel in comparison with docetaxel alone was demonstrated in a phase II clinical trial (47). Specifically, the primary endpoint of the study—median overall survival—was 9.4 months for the combination (selumetinib plus docetaxel) compared with 5.2 months for the control (placebo and docetaxel), although this difference was not statistically significant [hazard ratio (HR) for death 0·80, 80% CI 0·56–1·14; one-sided *p* = 0·21]. The median PFS was significantly improved in patients receiving selumetinib (5.3 vs. 2.1 months; HR for progression 0·58, 80% CI 0·42–0·79; one-sided *p* = 0·014) as was the response rate (37% vs. 0%; *p* < 0·0001). Subsequently, a subgroup analysis demonstrated that patients harboring G12V and G12C *KRAS* mutations appeared to experience higher RR and longer PFS for the combination arm, which was recently confirmed in preclinical mouse model (17).

Following this encouraging result, a further phase III clinical trial in *KRAS*-mutant lung cancer was conducted, which, however, failed to reproduce the significant benefit in patients treated with the combination compared with docetaxel alone (48). In this larger cohort trial, median PFS was 3.9 months with the combination group and 2.8 months with the control group (difference, 1.1 months; HR, 0.93 [95% CI, 0.77–1.12]; *P* = 0.44). Median overall survival was 8.7 and 7.9 months, respectively (difference, 0.9 months; HR, 1.05 [95% CI, 0.85–1.30]; *P* = 0.64). There is a marginally significant objective RR (20.1% in combination group vs. 13.7% in the control group; odds ratio, 1.61 [95% CI, 1.00–2.62]; *P* = 0.05). Whereas, the inconsistency with previous phase II trial results was unclear, a multitude of possible mechanisms, such as the aforementioned genomic (diverse point mutations and co-occurring alterations) and phenotypic (different EMT status) heterogeneity within the recruited patients, might be anticipated. In addition, chemotherapeutics used for combination treatment may need to be reconsidered in future studies, given that distinct amino acid substitutions of KRAS oncoproteins differed in their response to the commonly-used chemotherapy agents (9, 11). Nonetheless, these results did not rule out the effectiveness of this combination therapy in a subgroup of patients with *KRAS* mutations and deserved more detailed analysis, which might provide mechanistic information that facilitates patient stratification and prediction of potential responders.

Trametinib is another selective and potent MEK inhibitor that has been clinically approved for *BRAF* mutant cancers (mainly melanoma). Like selumetinib, the efficacy of trametinib, alone or in combination with docetaxel, has been evaluated in *KRAS*-mutant NSCLC. In a phase II trial, trametinib as a monotherapy showed RR and PFS similar to docetaxel in previously treated *KRAS*-mutant NSCLC (49). Another phase II study with *KRAS*-mutant NSCLC (n = 54, including 19 with G12C, 9 with G12D, 9 with G12A) documented a trend toward worse PFS (HR = 1.86, *p* = 0.06) and survival (HR = 1.80, *p* = 0.14) in G12C patients compared to non-G12C patients (50). Trametinib plus docetaxel had a RR of 33% and median survival of 11.1 months in patients with recurrent *KRAS*-mutant NSCLC. These results suggest that clinical responses to combined trametinib and docetaxel may differ between G12C and non-G12C patients.

#### Rewiring SHP2 Activities

SHP2 is a non-receptor protein tyrosine phosphatase, encoded by the *PTPN11* gene that is ubiquitously expressed. SHP2 is involved in signal transduction downstream of multiple growth factors, cytokine, and integrin receptors and, not surprisingly, functions as an essential player in oncogenesis (51, 52). Upon the activation of RTKs, the SH2 domain of SHP2 binds to the phosphorylated tyrosine residues and various substrates, such as RTKs, scaffolds and adaptor proteins, which enables SHP2 in its active state for enzymatical removal of phosphates (dephosphorylation) from the substrates.

Previous studies demonstrated that the adaptive reactivation of MAPK signaling in the presence of a MEK inhibitor was mediated by the loss of MAPK-dependent negative feedback loops and the consequent induction of RTKs signaling (26, 53). Recent studies in *KRAS*-mutant lung cancer (54, 55) and pancreatic adenocarcinoma (55), *KRAS*-amplified gastric carcinoma (56), and multiple other cancer models expressing mutant or wild-type *KRAS* (57, 58) revealed that the anti-tumor effect of MEK inhibitor treatment could be dramatically potentiated by concurrent SHP2 inhibition.

Specifically, targeting of MEK alone is frequently hampered by adaptive resistance, which is complex and context-dependent, and can involve activation of various RTKs, including ERBB family, AXL, PDGFR-α, or FGFR1 (26, 53, 59–62). Strikingly, SHP2, the key integrator of RTK-RAS signaling, was necessary for various contexts upon MEK blockade and required to re-establish MAPK signaling. Strong synergy was observed when SHP2 and MEK were simultaneously targeted, resulting in sustained inhibition of tumor growth in different cancer models.

These studies provided compelling evidence supporting further investigations of combining SHP2 and MEK inhibitors for patients with *KRAS*-mutant cancer. Fitting this tendency, the recent development of potent allosteric SHP2 inhibitors strengthens the interest in targeting SHP2 in cancer (63). A clinical trial (NCT03114319) investigating TNO155, an SHP2 inhibitor, in patients with *K*-/*N*-/*H*-*RAS, BRAF*, or *PTPN11* mutant tumors is ongoing.

#### Resurging Autophagy Inhibition

Potential therapeutic interventions to inhibit autophagy have been extensively studied in cancer. Tumor cells depend on macroautophagy to cope with oncogene-induced metabolic stress. Notably, in human cancer cell lines or tumors bearing *KRAS* mutations, high levels of basal autophagy were observed, making inhibition of autophagy therapeutically actionable in *KRAS*-driven tumors (64, 65).

Three simultaneously published studies signal a resurgence of interest to inhibit autophagy in *KRAS*-driven cancer (66–68). These studies indicated that upon the inhibition of the MAPK pathway, *KRAS*-mutant tumors depend on autophagy for survival and that, as a result, blocking this protective mechanism by concomitant inhibition of autophagy and MEK or ERK kinases is likely to be therapeutically beneficial in patients with *KRAS*-mutant pancreatic ductal adenocarcinoma, *NRAS*-mutant melanoma, and *BRAF*-mutant colorectal cancer (66, 67). More importantly, Kinsey et al. initiated off-label treatment for a patient with metastatic PDAC with trametinib and hydroxychloroquine, both of which have been clinically approved for other indications. They observed a striking disease response with a 50% reduction in tumor burden without toxicity (67).

Based on the intriguing findings, further clinical investigations are required to determine the benefits of combined MEK and autophagy for patients with activating mutations in the RAS–RAF–MEK–ERK pathway.

### Rewiring KRAS Activation

In normal cases, KRAS is activated in response to signaling from upstream RTKs. However, oncogenic *KRAS* mutations “lock” the protein in a constitutively active state, activating KRAS-dependent signaling in a RTKs-independent pattern. In line with this, clinical trials confirmed that patients with *KRAS*-mutant cancers generally have a poor response to the first generation of EGFR inhibitors, such as erlotinib and gefitinib, and the presence of *KRAS* mutations is used as a biomarker to exclude patients for EGFR inhibitor therapy (7, 8, 69).

However, recent studies have challenged this paradigm, which instead demonstrated that the activation of ERBB signaling was required for *KRAS*^G12D^-driven lung tumorigenesis in preclinical mice models and that pan-ERBB inhibition other than EGFR inhibition alone was strikingly effective to inhibit *KRAS*-mutant tumor growth and progression (61, 62).

In humans, the ERBB family contains HER1 (EGFR, ERBB1), HER2 (NEU, ERBB2), HER3 (ERBB3), and HER (ERBB4). Previous studies showed that ERBB3 activation was associated with resistance to MEK inhibition in *KRAS*-mutant NSCLC cells (26, 59). In a recent study by Kruspig et al. (61), the authors showed that multiple ERBB ligands (e.g., Areg, Ereg, Nrg3, Nrg4, and Hbegf) and receptors (for example, Erbb2 and Erbb3) were highly expressed in a *KRAS*^G12D^ mouse model. Neratinib, a multi-ERBB inhibitor (70, 71), almost completely suppressed the emergence of tumors. In sharp contrast, erlotinib failed to reproduce the same effect. Further mechanistic analyses of seemingly contradictory results revealed that ERBB activity establishes a feed-forward loop to amplify signaling through the core RAS-ERK cascade to sustain survival and proliferation in *KRAS*-mutant NSCLC. Indeed, pan-ERBB inhibition enhanced the potency of MEK inhibition *in vitro* and *in vivo*.

Similarly, an independent study by Moll et al. demonstrated a requirement for ERBB signaling to support the progression of *KRAS*^G12D^-driven lung cancer (62). In this study, an independent pan-ERBB inhibitor, afatinib, was used. Genetic mouse models revealed that EGFR deletion attenuates mutant KRAS activity and transiently reduces tumor growth. However, EGFR inhibition initiated a rapid resistance mechanism involving non-EGFR ERBB family members, which triggered a tumor escape mechanism. This provided an explanation, at least to some extent, for the poor unresponsiveness of *KRAS*-mutant lung cancer patients to the first-generation TKIs targeting EGFR alone. More importantly, afatinib blocked compensatory ERBB2 and ERBB3 activation, whereas erlotinib and gefitinib did not. Together, these studies suggested the therapeutic potential of pan-ERBB inhibitors in *KRAS*-driven tumors.

Notably, both studies revealed a requirement for simultaneous inhibition of multiple ERBB while targeting a single member of the family was not effective. These preclinical studies provide new insights into *KRAS*-driven tumorigenesis and bring new hope for *KRAS*-mutant lung cancer patients. Further trials, such as combined MEK with pan-ERBB inhibitors, are highly needed to determine the translational significance of the pan-ERBB inhibition strategy for patients, which can be easily and quickly conducted given that both inhibitors have been clinically approved (72, 73).

### Re-examining Downstream Partnership: CRAF (RAF1) but Not A-RAF or B-RAF

*KRAS* oncogenes signal through a cascade of downstream effectors, among which the most important one is the RAF/MEK/ERK cascade. The direct RAS downstream effectors within the RAF/MEK/ERK pathway are the RAF kinases, including A-, B-, and C-RAF. However, it is not well-known how these individual RAF kinases contribute to *KRAS*-mutant tumor initiation and development.

Recent studies showed that C-RAF rather than B-RAF plays a crucial role in mediating KRAS oncogenic signaling (74, 75). Targeting of C-Raf rather than of B-Raf kinase could recapitulate the effect of *Kras* ablation and effectively inhibit tumor development without inducing significant toxicities in mouse models of *Kras*/*Trp53*-mutant lung adenocarcinoma. This work suggested that distinct RAF kinases likely play different roles in mediating KRAS oncogenic signaling.

In a more recent study, ablation of *B*- or *C-RAF* was concomitant with *Kras*^G12V^ induction (76). C-Raf ablation completely prevented *Kras*-driven NSCLC without inducing deleterious effects, which, however, was not the case with B-Raf ablation, indicating that B-Raf is dispensable for Kras oncogenic signaling. Moreover, ablation of *C-Raf* did not affect Mek or Erk phosphorylation, suggesting that C-Raf-mediated Kras signaling is independent of the MAPK cascade. Further, the same group showed that combined inhibition of C-Raf and Egfr induced complete regression of pancreatic ductal adenocarcinomas in *Kras/Trp53*-driven GEM models and PDXs without apparent toxicity (77). Together, these studies provided compelling evidence that C-Raf, but not B-Raf or A-Raf, may mediate the oncogenic signaling in *KRAS*-driven cancer. More importantly, the therapeutic effect observed by ablation of *C-Raf* was likely due to disrupting the interaction of the C-Raf protein with other partners, such as BCL2, ASK1, MST2, and ROKα (76), whereas not via modulating MAPK cascade that is also essential for normal homeostasis. This might explain, to some extent, why the elimination of *C-Raf* did not induce systemic toxicity, in contrast to MEK inhibitors.

Notably, the therapeutic effect achieved by *C-Raf* ablation could not be reproduced by three C-Raf inhibitors that are designed to block the kinase activity other than the protein expression, confirming that the non-kinase activity of C-RAF instead of the conventional MAPK cascade is critical for the ability of *KRAS*-dependent oncogenic transformation. The striking finding of these studies, which is at odds with the currently ongoing efforts to develop C-RAF kinase inhibitors, implies, instead, the need for strategies to block C-RAF kinase-independent activities or induce its degradation.

### Revitalizing Chemotherapy

Currently, the platinum-based chemotherapy is still widely used for patients with *KRAS*-mutant lung cancer. However, the efficacy of chemotherapy is very limited, and durable response is generally short. Considerable efforts have been made to potentiate the efficacy of chemotherapy in *KRAS*-mutant cancer. Unfortunately, a recent phase III study has once again frustrated this attempt, which showed no additional survival benefit from combined MEK inhibitors compared to docetaxel alone (48).

Oncogenic KRAS signaling also involves PI3K-AKT-mTOR, via the interaction with the catalytic subunits of PI3K (78–80). Blocking RAS-mediated PI3K activation has also shown to inhibit the progression of *KRAS*-driven tumors. However, high toxicities of targeting PI3K, AKT or mTOR, in combination with MEK inhibitors, have prevented their approval for use in human patients (81–83).

We recently found that activation of mTOR signaling mediates a key resistance mechanism to chemotherapy in *KRAS*-mutant lung cancer (84). We observed exclusively hyperactivated mTOR signaling in lung cancer patient samples with *KRAS*-mutations but not in those carrying wild-type *KRAS*. Combined clinically approved mTOR inhibitor and chemotherapy showed a strong synergism in inhibiting proliferation of cancer cells harboring *KRAS*-mutation specifically. Additionally, the efficacy of this combination treatment correlates with the magnitude of mTOR activity induced by chemotherapy alone. Our results pinpoint a rational and readily translatable strategy that combines mTOR inhibitors with standard chemotherapy to treat *KRAS*-mutant lung cancer.

A recently published study provided novel hints to potentiate platinum-based chemotherapy in multiple cancer types (85). Jin et al. used multi-step kinome screens and identified MAST1, an AGC serine/threonine protein, as a key mediator of cisplatin resistance. The mechanistic study showed that MAST1 expression was increased in resistant cells and functioned as a MAP3K (MAPK kinase kinase), thereby activating MEK1 in cisplatin-resistant cells. Knockdown of *MAST1* re-sensitized the resistant cells to cisplatin *in vitro* and *in vivo*. Further investigations showed that cisplatin directly binds to cysteine 142 site of MEK1, restricting its access to C-RAF that typically phosphorylates MEK1. In this case, MAST1 took over C-RAF to re-activate MAPK cascade in cisplatin-resistant cells, and inhibition of MAST1 led to decreased MEK1 phosphorylation, explaining the effectiveness of targeting MAST1 in overcoming cisplatin resistance. More interestingly, MAST1 expression was shown to be specific to cisplatin rather than other similar agents (for instance, 5-fluorouracil) that interfere with DNA replication. This finding may be particularly relevant in the oncogenic *RAS/BRAF* setting, which mainly activates downstream MAPK signaling.

In a recent study (86), we reported that pemetrexed-resistant *KRAS*-mutant lung cancer cells assume a mesenchymal phenotype and cross-resist MEK inhibitors. Mechanistically, acquisition of resistance enables *KRAS*-mutant lung cancer cells to bypass canonical KRAS effectors but entail hyperactive AXL/eIF4E, increased protein turnover in the endoplasmic reticulum (ER), and adaptive activation of an ER stress-relief unfolded protein response survival pathway whose integrity is maintained by HSP90. In line with these mechanistic findings, HSP90 inhibitors synergistically enhance antitumor effects of pemetrexed and MEK inhibitors in multiple *in vitro* and *in vivo* models, validating a rational combination strategy to treat *KRAS*-mutant lung cancer.

### Reactivating Anti-tumor Immunity

Immune surveillance is generally dormant in cancer via dysregulation of immune checkpoints, such as the upregulation of the immunosuppressive protein PD-L1 for the evasion of the host immune system. Considerably convincing evidence has shown the importance of immune checkpoint blockade for treating various cancers (87). However, the therapeutic efficacy varies individually due to the high heterogeneity of tumors and the lack of reliable biomarkers to stratify the patients. Currently, limited biomarkers, such as PD-L1 expression (88) and tumor mutation load (TMB) (89–91), are clinically used to predict the immunotherapy benefit.

Interestingly, several preclinical studies suggested that tumors harboring *KRAS* mutations might be associated with a vulnerability to immunotherapies, in particular those with concomitant *TP53* mutations (29, 92, 93). Mechanistic studies indicated that oncogenic KRAS could stabilize PD-L1 mRNA through post-transcriptional modifications of the AU-rich element binding protein tristetraprolin (TTP) (94). Specifically, KRAS-MEK signaling contributes to phosphorylation and inhibition of TTP through the kinase MK2. In the same study, a high correlation between MAPK activation and elevated PD-L1 expression was observed in *KRAS*-mutant human lung and colorectal tumors.

A landmark trial (KEYNOTE-024) demonstrated that pembrolizumab was superior to chemotherapy in advanced NSCLC (potentially including patients with *KRAS*-mutations), among which more than 50% had high PD-L1 expression (95). Follow-up studies of the KEYNOTE-024 cohort revealed continuous survival benefit in patients treated with pembrolizumab as first-line monotherapy compared to those treated with chemotherapy (96).

Importantly, two remarkable phase III trials (KEYNOTE-189 and KEYNOTE-407) demonstrated that addition of pembrolizumab (Keytruda, anti-PD1) could significantly prolong the survival of NSCLC patients (45, 97). The promising results from these studies have led to the approval of pembrolizumab in combination with chemotherapy (carboplatin-pemetrexed) for metastatic, non-squamous NSCLC, and the approval of pembrolizumab in combination with chemotherapy (carboplatin and Taxol) for patients with squamous lung carcinoma, excluding those carrying *EGFR* or *ALK* mutations. Although patients with *KRAS*-mutant NSCLC were potentially included in the two studies, specific efficacy of the combination therapy on *KRAS*-mutant NSCLC remains to be investigated.

A recent systematic review and meta-analysis study, which integrated multiple randomized clinical trials, showed that immune checkpoint inhibitors significantly prolonged overall survival in the *KRAS*-mutant subgroup (HR, 0.65; 95% CI, 0.44–0.97; *P* = 0.03) but not in the *KRAS* wild-type one (HR, 0.86; 95% CI, 0.67–1.11; *P* = 0.24; interaction, *P* = 0.24) (98). Another meta-analysis study that incorporated 509 patients (138 of *KRAS*-mutant and 371 with *KRAS*-wild-type NSCLC) showed that, compared to docetaxel chemotherapy, immune checkpoint inhibitors improved overall survival in patients with previously-treated *KRAS*-mutant NSCLC (HR = 0.64 [95% confidence interval, 0.43–0.96], *P* = 0.03) (99), but not in patients with wild-type *KRAS* (HR = 0.88 [95% confidence interval, 0.68–1.13], *P* = 0.30). These results indicate that *KRAS* mutational status is a potential biomarker for survival benefits to immune checkpoint inhibitors. However, two other studies with advanced non-squamous NSCLC reported that the efficacy of immune checkpoint inhibitors is independent of *KRAS*-mutant status (100, 101). Thus, further studies with a stratification of *KRAS* genetic status are still needed.

Although, preclinical studies using immune-competent mouse models verified the promising efficacy of checkpoint blockade in the *Kras*-mutant setting (92, 102, 103), a majority of these studies relied on mouse models with a single genetic background, which limits the power to assess the potential influence of other co-occurring mutations (e.g., *STK11* alterations). Co-occurring genetic mutations, which can lead to differential downstream effectors engaged by mutant *KRAS*, have been reported to significantly affect the tumor immune signatures (25, 29, 31, 35) and responses to immunotherapy (30). This might explain why a pooled analysis of patient response data does not consistently support the association between benefits of immunotherapy and *KRAS* mutations (98–101).

## Conclusions

Targeting KRAS has represented a tremendous unmet clinical need. Nevertheless, the challenge of clinical treatment of *KRAS*-mutant cancers seems not to be insurmountable. Now, a new wave of attempts is motivated to target KRAS directly, which has long been considered undruggable. A striking response has been achieved with AMG510 in patients with *KRAS*^G12C^. New treatment strategies based on a deeper understanding of the pathobiology of oncogenic *KRAS*, such as abolishing C-RAF, blocking the universal rewiring of SHP2, and the protective autophagy in response to MEK inhibitors are highly promising with preliminary success in human patients. Conventional approaches, such as combined chemotherapy and mTOR inhibitors, as well as combined cisplatin and MAST1 inhibitors, are also encouraging but require further investigations in patients. The great success of immunotherapy has been witnessed in the treatment of patients with various tumors, but more evidence is required in cancer patients with *KRAS* mutations.

Given the presence of a variety of potent and specific chemicals, treating *KRAS*-mutant lung cancer remains a significant challenge, implying that the problem might be mechanism-related rather than the efficacy of targeting itself. A critical point is a high heterogeneity within *KRAS*-mutant tumors. To maximize the patient benefit, it cannot be more important than molecularly guided stratification on top of *KRAS* mutations.

## Author Contributions

HY and S-QL wrote the manuscript. RS reviewed the manuscript. R-WP outlined and revised the manuscript.

### Conflict of Interest

The authors declare that the research was conducted in the absence of any commercial or financial relationships that could be construed as a potential conflict of interest.
